# Possible Application of Ecological Momentary Assessment to Older Adults’ Daily Depressive Mood: Integrative Literature Review

**DOI:** 10.2196/13247

**Published:** 2020-06-02

**Authors:** Heejung Kim, Sunah Kim, Seong Sook Kong, Yi-Rang Jeong, Hyein Kim, Namhee Kim

**Affiliations:** 1 College of Nursing Yonsei University Seoul Republic of Korea; 2 Mo-Im Kim Nursing Research Institute Yonsei University Seoul Republic of Korea; 3 School of Nursing, College of Medicine Soonchunhyang University Cheonan Republic of Korea; 4 Samsung Medical Center Seoul Republic of Korea; 5 Severance Hospital Seoul Republic of Korea

**Keywords:** ecological momentary assessment, depression, aged, review

## Abstract

**Background:**

Ecological momentary assessment is a method of investigating individuals’ real-time experiences, behaviors, and moods in their natural environment over time. Despite its general usability and clinical value for evaluating daily depressive mood, there are several methodological challenges when applying ecological momentary assessment to older adults.

**Objective:**

The aims of this integrative literature review were to examine possible uses of the ecological momentary assessment methodology with older adults and to suggest strategies to increase the feasibility of its application in geriatric depression research and practice.

**Methods:**

We searched 4 electronic databases (MEDLINE, CINAHL, PsycINFO, and EMBASE) and gray literature; we also hand searched the retrieved articles’ references. We limited all database searches to articles published in peer-reviewed journals from 2009 to 2019. Search terms were “ecological momentary assessment,” “smartphone assessment,” “real time assessment,” “electronic daily diary,” “mHealth momentary assessment,” “mobile-based app,” and “experience sampling method,” combined with the relevant terms of depression. We included any studies that enrolled older adults even as a subgroup and that reported depressive mood at least once a day for more than 2 days.

**Results:**

Of the 38 studies that met the inclusion criteria, only 1 study enrolled adults aged 65 years or older as the entire sample; the remainder of the reviewed studies used mixed samples of both younger and older adults. Most of the analyzed studies (18/38, 47%) were quantitative, exploratory (descriptive, correlational, and predictive), and cohort in design. Ecological momentary assessment was used to describe the fluctuating pattern of participants’ depressive moods primarily and to examine the correlation between mood patterns and other health outcomes as a concurrent symptom. We found 3 key methodological issues: (1) heterogeneity in study design and protocol, (2) issues with definitions of dropout and adherence, and (3) variation in how depressive symptoms were measured with ecological momentary assessment. Some studies (8/38, 21%) examined the age difference of participants with respect to dropout or poor compliance rate. Detailed participant burden was reported, such as technical problems, aging-related health problems, or discomfort while using the device.

**Conclusions:**

Ecological momentary assessment has been used for comprehensive assessment of multiple mental health indicators in relation to depressive mood. Our findings provide methodological considerations for further studies that may be implemented using ecological momentary assessment to assess daily depressive mood in older adults. Conducting more feasibility studies focusing on older adults with standardized data collection protocols and mixed-methods research is required to reflect users’ experiences. Further telepsychiatric evaluation and diagnosis based on ecological momentary assessment data should involve standardized and sophisticated strategies to maximize the potential of ecological momentary assessment for older adults with depression in the community setting.

## Introduction

### Background

It is challenging to screen for and diagnose geriatric depression due to the atypical presentations of symptoms in older populations [1]. Consequently, geriatric depression often remains unrecognized in home care settings, even when the individual receives continuous home care service [2]. If health care providers use only instrument-based interviewing to screen for depression, they detect as few as one-third of depressed older adults [2]. It is important to diversify the available and implemented assessment methods to improve detection; thus, ecological momentary assessment (EMA) may help detect depressive mood more accurately.

EMA is a method of investigating individuals’ real-time experiences as they occur in their natural environment and situations over time [3]. EMA has a range of methodological strengths: (1) the “ecological” aspect represents real-world environments, allowing for increasing generalization with ecological validity; (2) the “momentary” aspect focuses on an individual’s current state, which may decrease retrospective bias and errors; and (3) the “assessment” aspect provides multiple data collection points over time and across situations [3,4].

EMA methods are used in psychological research [3]. Studies have used EMA methods to investigate individual affect [5-8], behavioral problems [9], and daily mental health symptoms [5,6,10]. For depression research, EMA has much to offer in terms of improving researchers’ understanding of depression because of advantages such as minimizing recall bias and detecting fluctuation of mood for a longer time [3,8]. Traditionally, researchers have relied on participants’ retrospective reports about their depressive mood; however, such recall data are subject to the vagaries of cognitive heuristics and the retrieval processes [3,8]. With EMA, participants may report their mood repeatedly over time, within a familiar real-life environment, rather than reporting recollections or being interviewed in a research or laboratory setting. Researchers or clinicians can gather more ecologically valid data, which reflect participants’ lifestyles or daily needs [3,8]. Thus, EMA can be used for diagnosing geriatric depression even without the screen instruments [11].

There are ongoing efforts to assure the validity, feasibility, and usability of EMA in individuals who report depressive symptoms [5,6]. For example, Hung et al [5] examined the validity and feasibility of smartphone-based EMA for Chinese patients with depression. Vachon et al [7] investigated changes in the psychological state of outpatients with major depressive disorder. Moore et al [12] found compatible psychometrics between traditional pen-and-paper and smartphone versions of EMA in emotionally distressed older adults. Among the growing body of EMA research in this area, many studies involved mixed age groups, such as middle-aged or older adults with younger participants [6,7,9,10,13-33]. Thus, it is unclear how older adults’ characteristics were reflected during data collection and interpretation using EMA because older adults with mental health problems frequently have decreased self-confidence and less motivation to use new technology [34].

### Objective

Very few studies have examined the feasibility of using EMA with older adults with depressive mood [34-36]. Thus, we believe that possible implications can be extracted from studies including older adults even when they are only part of the study population. Our integrative review aimed to (1) synthesize the current information regarding the possible application of EMA to older adults’ depression and (2) discuss the conceptual and methodological issues of EMA when considering further implementations in geriatric depression research and practice.

## Methods

This integrative review was based on a comprehensive literature search [37] and the Preferred Reporting Items for Systematic Reviews and Meta-Analyses guidelines [38].

### Search Strategy

For this integrative literature review, we conducted a literature search from June to July 2019. Based on the initial search, only 1 study [34] met the criterion that only older adults, aged 65 years or older, were enrolled. Thus, we decided to include studies that sampled adults aged 65 years or older as part of their samples; that is, both younger and older individuals might be included in the studies. We searched 4 electronic databases: MEDLINE (through PubMed), CINAHL, PsycINFO, and EMBASE. We searched the gray literature, including dissertations, conference proceedings (papers or abstracts), and editorials, in the Virginia Henderson International Nursing Library and CINAHL (exclusively focusing on gray literature). We performed an additional manual search using the Google Scholar online tool, based on an ancestry search of citation and reference lists obtained from articles we retrieved from the targeted databases.

The initial sets of search terms consisted of “ecological momentary assessment,” “smartphone assessment,” “real time assessment,” “electronic daily diary,” “mHealth momentary assessment,” “mobile-based app,” or “experience sampling method,” combined with “affect,” “mood,” and “emotion,” as well as “depress*” to reflect different relevant terms such as depression, depressed, or depressive (Multimedia Appendix 1).

#### Inclusion Criteria

Inclusion criteria were as follows: (1) studies enrolled at least some participants aged 65 years or older; (2) study participants reported EMA in the community setting; (3) studies measured momentary affect, such as depressive symptoms, depressed mood, negative affect, or negative emotion at least once a day for more than 2 days; (4) studies used certain types of instruments or devices to report participants’ momentary mood or scores, either electronic devices (eg, smartphone, personal digital assistant, or palm computer) or traditional pen-and-paper recording tools; (5) studies were published in English; and (6) studies were published between 2009 and 2019.

#### Exclusion Criteria

Exclusion criteria were as follows: (1) participants’ ages were not clearly reported or were determined to be less than 64 years using the available information regarding the study sample’s means, ranges, and proportion of age groups; (2) studies included a negligible proportion of older adults (eg, 65-69 years old) within a wide range of participant ages; (3) studies measured momentary affect such as depressive symptoms, depressed mood, negative affect, or negative emotion using non-EMA tools; and (4) studies were methodological studies comparing the reliability between traditional and electronic measurements of EMA.

If an article was a systematic review, Cochrane review, literature review, case study, or expert opinion, we used it as background information and examined the references to expand our manual literature search; however, we did not include such reviews in the analysis.

### Data Extraction, Analysis, and Synthesis

Four authors (YRJ, HYK, NHK, and HJK) initially screened titles and abstracts based on eligibility criteria and reviewed the full text of articles. These researchers had an acceptable level of agreement of over 95% regarding final selection of the articles. Four authors (YRJ, HYK, NHK, and HJK) extracted the data from the selected articles into an analysis table. Two authors (SK and SSK) validated and confirmed the analyzed data between articles and table entries for accuracy (99% verification).

## Results

### Characteristics of Selected Studies and Participants

**Figure 1:**
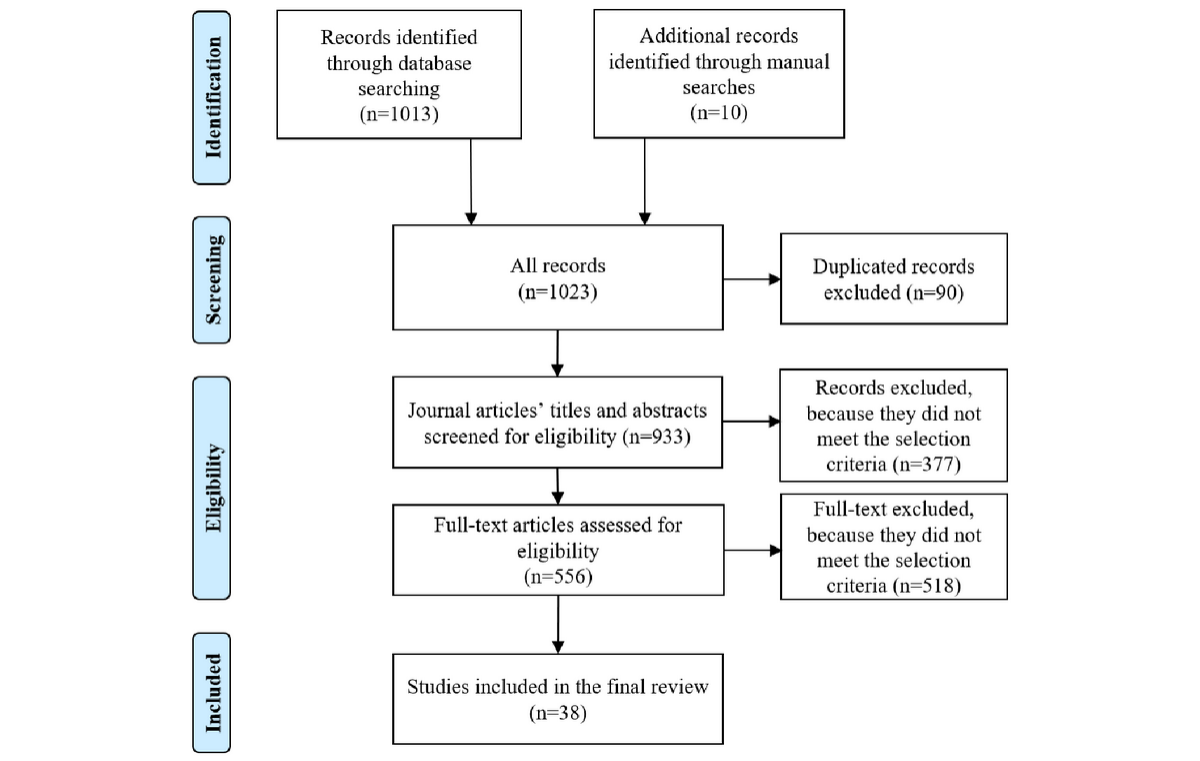
Preferred Reporting Items for Systematic Reviews and Meta-Analyses (PRISMA) flow diagram outlining the search and review process.

The searches retrieved 1013 records from the 4 databases and gray literature, and 10 records from the manual search. After screening the results against our eligibility criteria, we selected 38 studies for review that met our inclusion criteria (Figure 1). Multimedia Appendix 2 summarizes the characteristics of the 38 selected studies and their participants.

#### Study Aims and Design

All 38 studies applied a longitudinal design to observe depressive mood throughout EMA from several days to months to emphasize the benefit of multiple-timescale aspects of the study design. Most of the analyzed studies (18/38, 47%) were quantitative, exploratory (descriptive, correlational, and predictive), and cohort in design [6,9,10,15,18-20,22,24,25,28,32,33,36,39-42]. Approximately half of the studies used EMA primarily to assess depressive mood over time. However, the other half of the studies used EMA to examine other symptoms or health problems and collected information on depressive mood as concurrent or associated factors. Frequent aims of these studies were to (1) describe the fluctuating pattern of participants’ depressive mood and relevant characteristics and (2) identify concurrent and lagged association of the depressive mood with other psychological factors. Few studies examined predisposing situations and conditions that might influence a lagged effect of depressive mood. Other types of study designs were used to achieve different purposes. Two methodological studies [7,23] aimed to determine the validity, reliability, and user evaluation of EMA over time. Two studies applied a mixed-methods research approach [34,43] to assess perceived acceptability, adherence rates, and reasons for poor compliance or nonadherence to smartphone-based EMA in older adults or to examine participants’ qualitative responses.

#### Study Participants

Only 1 study enrolled adults aged 65 years or older as the entire sample [34]. The remainder of the reviewed studies used mixed samples of both younger and older adults, with ages ranging from 18 to 97 years. Some studies involved advanced age groups, such as middle-aged or older adults, with younger participants [6,7,9,10,13-33]. The sample size of surveyed studies ranged from 12 to 404, which varied according to study purpose, data collection method, and analysis plans. Some studies specifically aimed to enroll study participants with depressive mood and relevant mental health problems (7/38, 18%) [7,9,13,17,23,24,34], other physical health problems or diseases (8/38, 21%) [6,29,31,36,40,43-45], or chronic pain (2/38, 5%) [16,25], which may be related to depressive mood. Some studies aimed to enroll study participants who had an alcohol or smoking habit (4/38, 11%) [22,28,33,46] or obesity (2/38, 5%) [19,20]. However, several studies (15/38, 40%) did not specify any particular disease or condition [10,14,15,18, 21,26,27,30,32,39,41,42,47-49].

### Measures of Depression and Other Variables

Multimedia Appendix 2 and Multimedia Appendix 3 summarize the characteristics of EMA measurements used in the 38 selected studies.

#### Measures of Baseline Depression and Concurrent Characteristics

Most studies (22/38, 58%) examined depressive mood at baseline using a diverse array of clinically valid screening instruments or established diagnostic criteria. Several screening or diagnostic instruments were used: the Mini International Neuropsychiatric Interview [13,22,50]; Hamilton Rating Scale for Depression [13,29,51]; Beck Depression Inventory [7,13,48,52,53]; Patient Health Questionnaire (PHQ) [39,54]; Composite International Diagnostic Interview [40,55]; Montgomery-Asberg Depression Rating Scale [9,56]; *Diagnostic and Statistical Manual of Mental Disorders* (Fourth Edition) (DSM-IV) [7,17,23,34,57,58]; Structured Clinical Interview for the DSM-IV-Text Revision [10,25,59]; Patient-Reported Outcomes Measurement Information System (PROMIS) [34,60]; Center for Epidemiologic Studies Depression Scale (CES-D) [15,30,36,47,61]; Positive and Negative Affect Schedule (PANAS) [49,62]; Positive and Negative Syndrome Scale [24,63]; Hospital Anxiety and Depression Scale [16,43,64,65]; and Hamilton Depression Inventory [31,66]. The researchers of each study used this information to describe sample characteristics at baseline or included the data as a controlled variable in their analyses.

Some relevant characteristics were also examined along with depressive mood. Most studies included variables relating to momentary experiences, activities, or behaviors occurring before or at the time of report [6,9,13,16,17,21,23-32,39-44,47,48]. Some studies also included concurrent symptoms of depression such as fatigue [6,29,36,42,45], pain [6,16,25,31,36, 40,43,45,48,49], stress [9,10,18-22,26,28,29,32,33,41,42,44,46], anxiety [7,9,10,13,14,16,17,21,24-26,34,39-43,45,47], loneliness [14,20,23,24,27,31,42,46,47], or cognitive impairment [7,14,22, 27,34].

#### Measures of Momentary Depressive Mood

When employing EMA, most studies did not clearly define “momentary mood of depression,” and there was no consistency of operational definition across the 38 selected studies. Moreover, the depressive score was defined in diverse terms, such as depressive or negative mood and affect [6,7,9,10,13-29,31-34,36,39-48], sadness [9,15,17-22,24-30, 32,33,36,39,42,44,46,48,49], feeling down [13,14,23,47], or relevant symptoms of depression [6,29,31,34,45]. In some studies, depression was included as a set of subitems of global affect or mood [7,9,10,13,16,21,26,36,39-44], whereas other studies treated depressive scores separately from other measurements that might have been taken [6,30,31,34,45].

A single item was usually used to measure momentary depression in most studies (35/38, 92%), whereas only a few studies employed multiple items to assess symptoms of depression [31,34,36]. Most reporting mechanisms relied on a Likert scale (30/38, 79%), visual analog scale (6/38, 16%), or sliding bar scale (2/38, 5%) in the form of points or a sliding bar. Some of the surveyed studies (14/38, 37%) clearly stated that the question used to measure depression was extracted from widely used instruments, such as the PANAS [10,14,20,39,44,62], PANAS-Expanded Form [15,67], circumplex model of emotion [17,68], circumplex model of affect [19,26,69], PROMIS [34,70], Philadelphia Geriatric Center Positive and Negative Affect Rating Scale [36,71], CES-D-Revised [7,72], DSM-IV [29,57], or PHQ [31,54]. Those instruments were different from the measures used at baseline to screen participants’ depression diagnosis or initial status.

A range of different EMA devices were used to record momentary data. Of the 38 studies, 17 (45%) used computerized handheld devices [6,7,15,17,18,24,25,28,29,32,33,41,42, 44,45,47,49], whereas 14 studies (14/38, 37%) used smartphone or web-based EMA apps, or both [9,14,16,19-21, 23,26,30,34,39,40,43,46]. One study (1/38, 3%) employed telephone-based EMA via phone interview [36], and another relied on an automatic cell phone call [31]. One study (1/38, 3%) made a direct call and entered scores with an input keypad [22]. A few studies (3/38, 8%) used pen-and-paper–based EMA or booklets using a timekeeping mechanism [13,27,48]. One study (1/38, 3%) asked participants to record a diary via email, but their device was unclear [10].

Observation times varied widely across the studies. The number of repeated EMA measurements ranged from 1 to 10 per day, from 5 to 180 days. Thus, the total number of repeated EMA measurements ranged from 14 to 360. Several studies divided the EMA measurement period into 2 or more periods spaced over several months [18,20,21,23,26,30,34,47]; however, the other studies conducted EMA measurements within a consecutive period of days.

Most studies (36/38, 95%) provided detailed information about how EMA was applied within specific timing, except for 2 studies [30,43]. In several studies, participants received automatic notifications at fixed times [7,10,16,17, 25,32,39,42,44,46]. In other studies, the participant received an automatic notice at random within the researcher- or participant-designated time interval in a day [6,9,13, 14,21-23,29,36,40,41,48]. Other studies used a totally randomized time frame in a day [24,26,27,34,47,49]. Several studies used mixed methods, employing fixed or random and individualized time based on sleep and daily activities [15,18-20,28,31,33,45].

### Feasibility: Dropout, Adherence, and Subjective Evaluation

Many studies (34/38, 90%) defined dropout, compliance, adherence, or active usage. Multimedia Appendix 4 summarizes each concept’s definition and reported rate.

#### Definition and Overall Rate

Many studies defined dropout as participants who exhibited noncompliance during the study period [7,10,15,16,29,43]. One study applied liberal criteria by excluding those who never used EMA [10], whereas other researchers were relatively conservative, excluding even those who partially participated in the EMA but did not strictly follow the reporting protocols [7,15,29]. In addition, 11 studies (11/38, 29%) did not provide a clear operational definition of dropout, although they reported the rate of dropout. Based on these variously defined criteria and unclear information available across studies, the dropout rate among studies varied significantly, from 1.3% [10] to 25.9% [14].

Studies also reported mixed definitions of adherence and compliance. In 3 studies [7,9,45], these were defined as the degree of completion, calculated by dividing the number of completed ratings by the potential maximum number of required EMA observations. Other studies applied a specific required completion rate to classify whether a given participant adhered to or complied with EMA, such as 30% [34], 50% [6], or 100% [34]. Due to the inconsistency of adherence criteria, large variations in adherence rates were observed starting from 65.1% [9]. A total of 8 studies (8/38, 21%) examined age difference of dropout or poor compliance rate, but they reported that advanced age was not associated with different compliance or satisfaction with EMA reports [16,17,21,26,32,43,48,49]. However, 1 study [46] reported that older drinkers were more compliant than the younger group, specifically in the evening survey than in the morning ones.

#### Subjective Evaluation

Few studies reported specific reasons for participant dropout or lack of compliance when assessing EMA use. Those studies that did report this information noted that the most common reasons for withdrawing from study participation were loss of contact, acute health problems, adverse personal events, and decreasing interest or inability to complete the protocol [7,30]. Ramsey et al [34] reported detailed information regarding nonadherence specifically in older adults. The common themes of nonadherence were classified as (1) a technical problem or user error; (2) logistical mismatch with competing demands in the participant’s daily life; (3) health-related barriers, such as sensory, cognitive, or functional impairment; and (4) discomfort involved with carrying the device or completing EMA.

There was some researcher support to assure feasibility. For example, a previous study developed a mobile app that could run on both Android and iOS, allowing each study participant to choose the operating system with which he or she was most comfortable [5]. Because the app did not require an internet connection to record the score [5], study participants could log EMA reports anytime and anywhere. Alarming using a beeping prompt is very helpful to remind study participants of timely reports [13,24,25,27,33,42,44,48]. Specifically considering older adult participants’ potential sensory impairments, Ravesloot et al [6] provided additional assistance, such as a larger-format device or magnifiers for those with visual impairments and a stylus for those with dexterity problems.

## Discussion

### Principal Findings

This integrative review of the literature on EMA implementation in research provides some understanding of whether and how EMA may be feasibly applied with older adults when reporting daily depression or relevant conditions. Diverse conceptual and methodological issues should be considered when developing EMA protocols, and researchers should strive to establish rigorous validation procedures and clinical applications targeting older adults.

#### Heterogeneity in Study Design and Protocol

EMA protocols are more complicated and time consuming than traditional one-time surveys. The 38 studies reviewed used a great diversity of protocols, with little consistency in the methodologies. Some studies used new protocols developed by the researchers [29] or previously established ones from large-scale studies [9]. Protocol content encompassed optimal frequency, duration, and interval of data collection, as well as the device employed to collect the data. Based on the technology acceptance model framework [73], EMA systems should be very simple, reducing users’ cognitive errors and enhancing response accuracy. Thus, single items were most frequently used for EMA as longitudinally intensive designs because repeated use of multi-item scales may be impractical for a depressed sample [74], specifically older adults. However, a single question has lower construct validity than multiple questions. Thus, researchers should make a careful decision when choosing fewer items from preexisting measures to assess geriatric depression.

To assure ecological validity, most studies adopted an individualized protocol, which increased the heterogeneity of the studies. EMA reporting time within a day should consider each individual’s lifestyle, preference, and convenience [7,75] to minimize interrupting participants’ daily lives [7,9], such as sleep [9]. It is important to collect information on participants’ current engagement in daily life activities, location, and social context or event when engaging in EMA [6,9,30,31,39,44]. This information could be used to differentiate normal patterns in a participant’s daily life versus abnormal data that occur in specific situations or environments [34].

Diverse theories were used in the EMA studies identified in our review [14,22,25,26,30,32,44], such as the social support theoretical model [44]; the Intraindividual Study of Affect, Health, and Interpersonal Behavior [30]; the strength and vulnerability integration theory [32]; the communal coping model [25]; the social action theory [22] and the dynamic model of relapse [22]; and the cognitive appraisal theory [14]; as well as a mix of hedonic motivation [26] and operant conditioning [26]. It is important to conceptualize complex psychosocial processes measured by multiple-time assessment and modeling. Any theory employed to underpin research in this area should be modified appropriately, focusing on psychological aspects of specific age groups to promote clinical practice emphasizing socioenvironmental factors. This theoretical effort may promote the context-sensitive development of appropriate study protocols, taking into account individual and population needs.

#### Issues With Definitions of Dropout and Adherence

Some degree of dropout and nonadherence occurred with wide variability. Common reasons for dropout or lack of adherence were associated with technical, logistical, physical, and cognitive problems, similar to issues reported in previous studies [34,76], rather than with advanced age. Thus, the selected studies carefully screened participants who might be lacking in technological aptitude based on medical and functional conditions that could inhibit accurate EMA reporting rather than excluding participants based solely on advanced age [9,10,16,18,22,25,29,32,34,36,40,45-47]. Some studies excluded individuals from participation when they had (1) severe cognitive impairment, such as dementia; (2) severe symptoms of cognitive or emotional disturbances; or (3) other psychiatric diagnoses (eg, schizophrenia or substance abuse) requiring intensive treatment and hospitalization. EMA studies with depressed older adults should be preceded by comprehensive assessment, including physical examination, cognitive and functional tests, and an intensive personal interview regarding health conditions. In addition, it is important to ensure ease, comfort, satisfaction, and accessibility when using an EMA device, app, or system [6]. Familiarity may be increased by developing a user-friendly app’s features, visual layout, or system based on users’ experience [5].

#### Variation in How Depressive Symptoms Were Measured With EMA Devices

EMA was applied to measure daily depressive mood using electronic devices such as smart devices, computerized handheld devices, or telephones. Most studies used an electronic diary format rather than personal direct interviewing [36], aligning with the dramatic development of information and communication technology in the field. Because EMA requires multiple self-reporting instances in daily life, electronic and smart devices may be more suitable for this type of research; they are portable and easy to use in a range of situations and at various times [7].

However, device training should be provided to participants, particularly in light of diverse participant backgrounds and technological experience [77]. Although none of the 38 selected studies reported details about the training procedure itself, the participants were instructed on how to respond to prompts regarding their psychological state of momentary mood [6,7,13,15,17-20,23-26,28-34,40-42,45,47-49]. Researchers usually checked participants’ understanding of EMA report, functions of the device, or early compliance through daily review [9,17,22,28,31-34,44,49]. After that, some researchers provided the participants with a device practice opportunity under a researcher’s supervision [31,32,34] or rental of the device [6,20,23,24,28,29,33,40,46,49]. Several studies provided a training guidebook [6,20,25,40,49]. To apply EMA in practice, it is important that health care professionals learn how to use information and communication technology devices on their own, then teach the techniques to their patients or patients’ families to monitor older adults’ symptoms [78].

### Implications for Research and Practice

Based on our study findings, EMA could enhance health care professionals’ ability to detect changes in patient-reported emotions (eg, mindfulness, depression, and anxiety) in comparison with standard assessment instruments [12]. However, more research should be conducted with older adult participants to confirm the technique’s suitability in this context. It may be difficult to generalize our review findings due to mixed samples of diverse age groups. However, the identified methodological challenges should be overcome by further studies in both clinical and community settings. Specifically, future research should investigate factors that influence adherence and complete use of EMA when this technique is used as a self-monitoring system reflecting clinical and ecological validation along with the value of technology.

Health care professionals should prepare themselves to use EMA by engaging in multimodal training prior to performing their role; moreover, they should deliver timely device training adapted to meet the needs of diverse study participants [79]. Health care providers typically learn via hands-on experience based on a trial-and-error system or postprofessional certification programs [80]. To enhance standardization and rigor, as well as to improve implementation, health care professionals should be made aware of the importance of EMA, as well as the methodological challenges of appropriately implementing EMA when dealing with geriatric depression. Our study findings could be fundamental to developing user-centered EMA strategies for older users.

### Limitations

This study had several limitations. First, we initially attempted to include only adults aged 65 years or older; however, only 1 study met this criterion [34]. Thus, this integrative literature review included studies that accepted participants aged 18 years or older, including older adults. This limitation precluded our study findings to apply directly to older adults only. Our study findings may be appropriate for studies including both younger and older adults by using methodological approaches that specifically accommodate or are tailored to older adults. In the future, EMA research specifically targeting older adults needs to be conducted, and further analysis is required. Second, due to limited age-related information in the surveyed studies, we were unable to perform quantitative examinations to assess how proportions of older adults relate to adherence or dropout rates.

### Conclusions

EMA is becoming an increasingly popular approach to assess depressive symptoms, and this technique has particular clinical value with older adults. This integrative literature review provides a distinctive understanding of the feasibility of employing EMA to investigate depressive mood among older adults. In the studies under review, EMA was used to examine the correlation between pattern of mood and other health outcomes and to investigate changes in this pattern caused by triggers in terms of effects on treatment and reported symptoms. Further research and guidelines for clinical practice should be developed in consideration of how to evaluate participants’ competence to complete EMA; how to prevent dropout, nonadherence, and data incompleteness; how to use valid measures of momentary depressive mood; how to standardize EMA protocols; and how to ensure sufficient sample sizes. Our study findings support the need to overcome these methodological challenges and facilitate future research demonstrating the clinical implications of EMA, and suggest the next step toward the successful development of ecological momentary interventions for older adults with depression.

## Appendix

Multimedia Appendix 1 Search strategy overview.

Multimedia Appendix 2 Characteristics of the 38 selected studies.

Multimedia Appendix 3 Summary of the characteristics of ecological momentary assessment used in the 38 studies.

Multimedia Appendix 4 Definitions and rates of dropout, adherence, and compliance used in the selected studies.

## Abbreviations

CES-D: Center for Epidemiologic Studies Depression Scale
DSM-IV: Diagnostic and Statistical Manual of Mental Disorders (Fourth Edition)
EMA: ecological momentary assessment
PANAS: Positive and Negative Affect Schedule
PHQ: Patient Health Questionnaire
PROMIS: Patient-Reported Outcomes Measurement Information System
