# Supplementary material for: Possible Application of Ecological Momentary Assessment to Older Adults’ Daily Depressive Mood: Integrative Literature Review
Source: JMIR Ment Health. 2020 Jun 2;7(6):e13247. doi: 10.2196/13247 (PMC7298638; doi:10.2196/13247)
Supplement: Multimedia Appendix 1 [file mental_v7i6e13247_app1.docx]

Multimedia Appendix 1. Search strategy overview

| Search | | Query | Item found |
| --- | --- | --- | --- |
|  | |  |  |
| **MEDLINE** | |  |  |
|  | #1 | (“ecological momentary assessment”[Mesh] OR smartphone assessment OR real time assessment OR electronic daily diary OR mHealth momentary assessment OR mobile-based app OR experience sampling method) | 20719 |
|  | #2 | ("Affect"[Mesh] OR "Affective Symptoms"[Mesh] OR affection OR affect* OR "Mood Disorders"[Mesh] OR mood OR moods OR "Emotions"[Mesh] OR emotion OR emot*) | 1094523 |
|  | #3 | ("Depressive Disorder"[Mesh] OR "Depressive Disorder, Major"[Mesh] OR "Depression"[Mesh] OR depress*) | 219790 |
|  |  | #1 AND #2 AND #3 | 510 |
| **CINAHL** | |  |  |
|  | #1 | (“ecological momentary assessment”[Mesh] OR smartphone assessment OR real time assessment OR electronic daily diary OR mHealth momentary assessment OR mobile-based app OR experience sampling method) | 726 |
|  | #2 | ("Affect"[Mesh] OR "Affective Symptoms"[Mesh] OR affection OR affect* OR "Mood Disorders"[Mesh] OR mood OR moods OR "Emotions"[Mesh] OR emotion OR emot*) | 223188 |
|  | #3 | ("Depressive Disorder"[Mesh] OR "Depressive Disorder, Major"[Mesh] OR "Depression"[Mesh] OR depress*) | 76239 |
|  |  | #1 AND #2 AND #3 | 32 |
| **PsycINFO** | |  |  |
|  | #1 | (“ecological momentary assessment”[Mesh] OR smartphone assessment OR real time assessment OR electronic daily diary OR mHealth momentary assessment OR mobile-based app OR experience sampling method) | 4579 |
|  | #2 | ("Affect"[Mesh] OR "Affective Symptoms"[Mesh] OR affection OR affect* OR "Mood Disorders"[Mesh] OR mood OR moods OR "Emotions"[Mesh] OR emotion OR emot*) | 359048 |
|  | #3 | ("Depressive Disorder"[Mesh] OR "Depressive Disorder, Major"[Mesh] OR "Depression"[Mesh] OR depress*) | 135239 |
|  |  | #1 AND #2 AND #3 | 199 |
| **EMBASE** | |  |  |
|  | #1 | ('ecological momentary assessment[mesh]':ab,ti OR 'smartphone assessment':ab,ti OR 'real time assessment':ab,ti OR 'electronic daily diary':ab,ti OR 'mhealth momentary assessment':ab,ti OR 'mobile-based app':ab,ti OR 'experience sampling method':ab,ti) AND [1-1-2009]/sd NOT [5-7-2019]/sd | 1401 |
|  | #2 | ('affect [mesh]':ab,ti OR 'affective symptoms [mesh]':ab,ti OR affection:ab,ti OR affect*:ab,ti OR 'mood disorders [mesh]':ab,ti OR mood:ab,ti OR moods:ab,ti OR 'emotions [mesh]':ab,ti OR emotion:ab,ti OR emot*:ab,ti) AND [1-1-2009]/sd NOT [5-7-2019]/sd | 1420760 |
|  | #3 | ('depressive disorder [mesh]':ab,ti OR 'depressive disorder, major [mesh]':ab,ti OR 'depression [mesh]':ab,ti OR depress*:ab,ti) AND [1-1-2009]/sd NOT [5-7-2019]/sd | 310503 |
|  |  | #1 AND #2 AND #3 | 68 |
