# Supplementary material for: Possible Application of Ecological Momentary Assessment to Older Adults’ Daily Depressive Mood: Integrative Literature Review
Source: JMIR Ment Health. 2020 Jun 2;7(6):e13247. doi: 10.2196/13247 (PMC7298638; doi:10.2196/13247)
Supplement: Multimedia Appendix 2 [file mental_v7i6e13247_app2.docx]

Multimedia Appendix 2. Characteristics of the 38 selected studies.^a-u^

| First author,  year | Primary aims to target depression | Design | Sample | | | Baseline depression assessed with |
| --- | --- | --- | --- | --- | --- | --- |
|  |  |  | Age | Country | Number^a^ of participants enrolled |  |
|  |  |  |  |  |  |  |
| Crowe, 2019 [13] | Yes | • Quantitative  • Correlational  • Case-control  • Cohort | • Range: 18 to 70 | Ireland | 31 MDD^b^ group and 33 healthy control group | MINI^c^ Plus 5.0.0;  HAM-D^d^;  BDI^e^ |
| de Hoog, 2019 [14] | Yes | • Quantitative  • Correlational  • Cohort | • Men:  Mean ± SD^f^ = 49.8 ± 17.3  • Women: Mean ± SD = 45.5 ± 14.1  • Range: 18 to 82 | Netherlands | 85 adults | None |
| Mogle, 2019 [15] | Yes | • Quantitative  • Correlational and predictive  • Cohort  • Secondary data analysis | • Mean ± SD = 49.5 ± 16.81  • Range: 20 to 79  • Adults > 65: 16% | USA^g^ | 180 adults | CES-D^h^ |
| Hooker, 2018 [39] | No | • Quantitative  • Correlational and predictive  • Cohort  • Secondary data analysis | • Mean ± SD = 43.4 ± 10.7  • Range: 30 to 72 | USA | 80 inactive adults | PHQ^i^-8 |
| Jamison, 2018a [43] | No | • Mixed method  • Case-control  • Cohort | • Mean ± SD = 63.0 ± 7.8  • Range: 40 to 72 | USA | 69 women with hand pain related to osteoarthritis | HADS^j^ |
| Jamison, 2018b [16] | No | • Quantitative  • Correlational  • Cohort | • Mean ± SD = 46.7 ± 12.9  • Range: 18 to 79 | USA | 90 chronic pain patients | HADS |
| Kuerbis, 2018 [46] | No | • Quantitative  • Correlational  • Cohort  • Secondary data analysis | • Adults ≤ 49:  Mean ± SD = 35.8 ± 7.9  • Adults ≥ 50: Mean ± SD = 57.5 ± 5.4  • Total:  Mean ± SD = 43.2 ± 12.5  • Range: 20 to 73 | USA | 139 adults with a current alcohol use disorder | None |
| Lamers, 2018 [17] | Yes | • Quantitative  • Correlational  • Case-control  • Cohort  • Secondary data analysis | • Mean ± SD = 48.2 ± 16.5  • Range: 18 to 84 | USA | 33 BPI^k^, 37 BPII^l^, 116 MDD, 36 anxiety disorders without a mood disorder, and 65 controls without the conductions | Interviewed based on DSM-IV^m^-Texas Revision |
| Neubauer, 2018 [18] | Yes | • Quantitative  • Correlational and predictive  • Cohort | • Mean ± SD = 49.7 ± 17.1  • Range: 20 to 79 | USA | 180 adults | None |
| Paolillo, 2018 [40] | No | • Quantitative  • Correlational and predictive  • Case-control  • Cohort | • Mean ± SD = 57.5 ± 6.1  • Range: 50 to 74 | USA | 22 HIV^n^-seropositive and 13 HIV-seronegative | Interviewed based on CIDI^o^ |
| van Knippenberg, 2018 [47] | No | • Quantitative  • Correlational  • Cohort  • 3-armed RCT^p^ | • Mean ± SD = 72.1 ± 8.4 | Netherlands | 76 informal caregivers of community-dwelling people with dementia | CES-D |
| Elliston, 2017 [19] | No | • Quantitative  • Correlational and predictive  • Cohort | • Mean ± SD = 31.81 ± 14.87  • Range: 19 to 73 | Australia | 51 adults with overweight and obesity | None |
| Forman, 2017 [20] | No | • Quantitative  • Correlational and predictive  • Cohort  • Secondary data analysis | • Mean ± SD = 51.81 ± 9.76  • Range: 18 to 70 | USA | 189 overweight adults | None |
| Liao, 2017 [21] | Yes | • Quantitative  • Correlational  • Cohort  • Secondary data analysis | • Mean ± SD = 40.2 ± 8.6  • Range: 28 to 71 | USA | 71 low-active adult dog owners | None |
| Maisto, 2017 [22] | Yes | • Quantitative  • Correlational and predictive  • Cohort  • Secondary data analysis | • Mean ± SD = 34.4 ± 11.79  • Range: 19 to 76 | USA | 119 outpatients with alcohol use disorder | Interviewed  based on  MINI 6.0/eMINI |
| Verhagen, 2017 [23] | No | • Quantitative  • Correlational  • Cohort | • Mean age = 48.7 ± 13.9  • Range: 18 to 73 | Netherlands | 75 outpatients at mental health service | DSM-IV-Texas Revision |
| Depp, 2016 [9] | Yes | • Quantitative  • Correlational and predictive  • Cohort  • Secondary data analysis | • Mean ± SD = 46.9 ± 11.8  • Range: 18 to 72 | USA | 41 outpatients with BPI or BPII | Montgomery Asberg Depression Rating Scale |
| Eldahan, 2016 [10] | Yes | • Quantitative  • Correlational and predictive  • Cohort  • Secondary data analysis | • Mean ± SD = 37 ± 11.5  • Range: 18 to 73 | USA | 376 gay or bisexual men | Interviewed  based on  SCID^q^ |
| Paterson, 2016 [44] | Yes | • Quantitative  • Correlational  • Cohort  • Secondary data analysis | • Range: 51 to 73 | England | 12 men with prostate cancer within 6 months of diagnosis | None |
| Ramsey, 2016 [34] | No | • Mixed method  • Correlational  • Cohort  • Secondary data analysis | • Mean ± SD = 71.9 ± 5.4  • Eligible when 65yrs old or more | USA | 103 older adults with subjective cognitive and emotional difficulties | DSM-IV; PROMIS-SF^r^ |
| Ravesloot, 2016 [6] | Yes | • Quantitative  • Correlational and predictive  • Cohort | • Mean ± SD = 55.7 ± 11.2  • Range: 19 to 75 | USA | 149 community participants with disability | None |
| Smith, 2016 [36] | Yes | • Quantitative  • Correlational and predictive  • Cohort | • African American:  Mean ± SD = 61.6 ± 7.6  • Non-Hispanic whites:  Mean ± SD = 63.8 ± 9.8  • Range: 49 to 97 | USA | 39 African Americans and 81 non-Hispanic whites with osteoarthritis | CES-D |
| Vachon, 2016 [7] | Yes | • Quantitative  • Cohort | • Mean ± SD = 51.4 ± 9.6  • Range of inclusion criteria: 18 to 70 | France | 28 outpatients with MDD | DSM-IV-Texas Revision; BDI-II |
| Vasconcelos e Sa, 2016 [24] | Yes | • Quantitative  • Correlational and predictive  • Cohort | • Patient:  Median = 26  Range: 19 to 51  • Relative:  Mean ± SD =  52 ± 13.9  Range: 22 to 79 | England | 25 patients experiencing psychosis and their closest 23 relatives | Interviewed  based on  PANSS^s^ |
| Burns, 2015 [25] | No | • Quantitative  • Correlational and predictive  • Cohort | • Patient:  Mean ± SD = 46.3 ± 12.1  • Spouse:  Mean ± SD = 45.96 ± 13.2  • Range of inclusion criteria: 18 to 70 | USA | 113 married couples (one spouse with chronic low back pain) | Interviewed  based on  SCID-IV/NP^t^ |
| Droit-Volet, 2015 [48] | No | • Quantitative  • Correlational  • Case-control  • Cohort | • Young group:  Mean ± SD = 21.75 ± 1.70  • Elderly group  Mean ± SD = 72.75 ± 5.27  Range: 69 to 75 | France | 29 participants | BDI |
| Dunton, 2015 [26] | Yes | • Quantitative  • Correlational  • Cohort  • Secondary data analysis | • Mean ± SD = 40.5 ± 9.5  • Range: 27 to 73 | USA | 116 healthy adults | None |
| Floridou, 2015 [27] | No | • Quantitative  • Correlational  • Cohort | • Range: 18 to 72 | England | 40 participants | None |
| Epler, 2014 [28] | No | • Quantitative  • Correlational and predictive  • Cohort  • Secondary data analysis | • Mean ± SD = 23.48 ± 7.45  • Range: 18 to 70 | USA | 404 frequent drinkers | None |
| Mazure, 2014 [29] | Yes | • Quantitative  • Cohort | • Mean ± SD = 59.30 ± 13.34  • Range: 28 to 83 | France | 48 post-stroke patients | HAM-D |
| Ram, 2014 [30] | Yes | • Quantitative  • Predictive  • Cohort | • Mean ± SD = 47.64 ± 18.85  • Range: 19 to 89  • 50-64yrs: 27.3%; 65yrs old or more: 20% | USA | 150 healthy adults | CES-D |
| Wolf, 2014 [31] | No | • Quantitative  • Correlational  • Cohort | • Mean ± SD = 52.03 ± 11.34  • Range: 19 to 72 | USA | 154 adults with fibromyalgia | Hamilton Depression Inventory |
| Scott, 2013 [32] | Yes | • Quantitative  • Correlational and predictive  • Cohort  • Secondary data analysis | • Mean ± SD = 48.86 ± 19.29  • Range: 20 to 81 | USA | 201 adults | None |
| Kööts, 2011 [49] | Yes | • Quantitative  • Correlational  • Case-control  • Cohort | • Older group:  Mean ± SD = 68.2 ± 5.5  Range: 61 to 84  • Undergraduate student group:  Mean ± SD = 21.3 ± 1.0  Range: 19 to 23 | Estonia | 110 participants | Estonian version of PANAS^u^ |
| Piasecki, 2011 [33] | No | • Quantitative  • Correlational and predictive  • Case-control  • Cohort | • Mean ± SD = 25.1 ± 8.5  • Range: 18 to 70 | USA | 259 current smokers and 145 current drinkers who did not smoke | None |
| Hachizuka, 2010 [45] | No | • Quantitative  • Correlational  • Cohort | • Mean ± SD = 67.1 ± 9.2  • Range: 55 to 91 | Japan | 18 patients with terminal cancer receiving in-home palliative care | None |
| Poulin, 2010 [41] | No | • Quantitative  • Correlational and predictive  • Cohort | • Mean ± SD = 71.47 ± 10.6  • Range: 35 to 89 | USA | 73 caregivers providing full-time home care to their ailing spouse | None |
| Dunton, 2009 [42] | Yes | • Quantitative  • Correlational and predictive  • Cohort | • Mean ± SD = 60.65 ± 8.22  • Range: 50 to 76 | USA | 28 healthy and community-dwelling adults | None |

^a^Meaning of N: the number of study participants who were recruited first.

^b^MDD: Major Depressive Disorder.

^c^MINI: Mini International Neuropsychiatric Interview.

^d^HAM-D: Hamilton Rating Scale for Depression.

^e^BDI: Beck Depression Inventory.

^f^SD^:^ Standard Deviation.

^g^USA: United States of America.

^h^CES-D: Center for Epidemiologic Studies Depression Scale.

^i^PHQ: Patient Health Questionnaire.

^j^HADS: The Hospital Anxiety and Depression Scale.

^k^BPI: Bipolar I disorder.

^l^BPII: Bipolar II disorder.

^m^DSM-IV: Diagnostic and Statistical Manual of Mental Disorders, Fourth Edition.

^n^HIV: Human Immunodeficiency Virus.

^o^CIDI: Composite International Diagnostic Interview.

^p^RCT: Randomized Controlled Trial.

^q^SCID: Structured Clinical Interview for the DSM-IV-TR.

^r^PROMIS-SF: Patient Reported Outcomes Measurement Information System, Short Form.

^s^PANSS: Positive and Negative Syndrome Scale.

^t^SCID-IV/NP: Structured Clinical Interview for DSM-IV Axis I Disorders - Non-Patient Edition.

^u^PANAS: Positive and Negative Affect Schedule.
