# Supplementary material for: Possible Application of Ecological Momentary Assessment to Older Adults’ Daily Depressive Mood: Integrative Literature Review
Source: JMIR Ment Health. 2020 Jun 2;7(6):e13247. doi: 10.2196/13247 (PMC7298638; doi:10.2196/13247)
Supplement: Multimedia Appendix 3 [file mental_v7i6e13247_app3.docx]

Multimedia Appendix 3. Summary of the characteristics of EMA^a^ used in the 38 studies.^a-h^

| First author, year | How to measure depressive mood | Other variables | Type of device | Timing | Maximum time allowed to enter the data | Times  /day | Days (consecutive) |
| --- | --- | --- | --- | --- | --- | --- | --- |
|  |  |  |  |  |  |  |  |
| Crowe, 2019 [13] | • One item (down) among 6 NA^b^  • Self-reporting question based on 7-point Likert scale | • Self-esteem  • Suicidality  • Tiredness  • PA^c^ (happy, relaxed, interested, and enjoying myself)  • Other 5 NA (irritated, anxious, tense, ashamed, and guilty) | • Wristwatch (alarm) and pen-and-paper (record) | • Quasi-random basis with 1.5 hours block from 8am to 10pm30 | 25 minutes | 10 | 6 |
| de Hoog, 2019 [14] | • One item (down) among 6 NA  • Self-reporting question based on 7-point Likert scale | • News perception, valence, and type  • Cognitive appraisal (personal relevance)  • Neuroticism  • Extraversion  • PA (cheerful, relaxed, and content)  • Other 5 NA (insecure, lonely, anxious, irritated, and guilty) | • Mobile devices with application (LifeData) | • Quasi-random basis with 1.5 hours average block from 8am to 10pm30 | 60 minutes | 5 | 10 |
| Mogle, 2019 [15] | • One item (sad) among 4 NA  • Self-reporting question based on 7-point Likert scale | • Memory lapse  • PA (happy, enthusiastic, content, and excited)  • Other 3 NA (tense, upset, and disappointed) | • Palmtop computer completed daily diary work | • Mixed: (1) Quasi-random basis 5 prompts with 3 hours block (not analyzed)  (2) Unclear with a morning report (not analyzed)  (3) Once from 7pm to 3am as an evening report | Not described | 7 | 7 |
| Hooker, 2018 [39] | • One item (sad) among 5 negative mood  • Self-reporting question based on 5-point Likert scale | • Meaning salience  • Physical activity  • Positive mood (relaxed, proud, excited, appreciative, enthusiastic, happy, satisfied, and curious)  • Other 4 negative mood (sluggish, afraid, anxious, and angry) | • Email  • Web-based application (Research Electronic Data Capture) for online daily diary | • Fixed basis at 8pm | Not described | 1 | 28 |
| Jamison, 2018a [43] | • Mood (depressed and anxious)  • Single Self-reporting question based on 10-point VAS^d^ | • Ratings of pain  • Pain interfered with daily activities  • Pain interfered with sleep  • Whether things had changed for better or worse | • Smartphone with application | • Unclear | Not described | 1 | 3-month |
| Jamison, 2018b [16] | • Mood (depressed and anxious)  • Single self-reporting question based on 10-point VAS | • Ratings of pain  • Pain interfered with daily activities  • Pain interfered with sleep  • Whether things had changed for better or worse  • Daily activity | • Smartphone with application  • Fitbit | • Fixed basis at the pre-determined time by each participant | Not described | 1 | 6-month |
| Kuerbis, 2018 [46] | • One item (sad) among 2 mood that reflect mood over the last hour  • Self-reporting question based on 8-point Likert-type scale | • Drinking outcomes  • Loneliness  • Boredom  • Stress  • Poor sleep quality  • Social influence  • Commitment  • Confidence  • Alcohol salience  • Other one mood that reflect mood over the last hour (happy) | • Smartphone  • Online daily survey using the web browser | • Fixed basis at the pre-determined time by each participant | Not described | 2 | 7 |
| Lamers, 2018 [17] | • One item (sad) among 2 negative mood that reflect mood states  • Self-reporting question based on 7-point Likert-type scale | • Daily event  • Other one negative mood that reflect mood states (anxious) | • PDA^e^ | • Fixed basis at the pre-determined time by each participant with 2-6 hours interval | Not described | 4 | 14 |
| Neubauer, 2018 [18] | • One item (sad) among 4 NA  • Self-reporting question based on 7-point Likert scale | • Stressor exposure  • Stressor anticipation  • Neuroticism  • Intrusive thoughts  • Other 3 NA (tense, upset, and disappointed) | • Palmtop computer | • Mixed: (1) Quasi-random basis 5 prompts with 2-3 hours block  (2) A morning report and an evening report were initiated by participants | Not described | 7 | 21  (7 consecutive days * 3 cycles) |
| Paolillo, 2018 [40] | • One item (depression) among 3 mood  • Self-reporting question based on 5-point Likert scale | • Substance use  • Pain  • Other 2 mood (anxiety and happiness) | • Smartphone | • Quasi-random basis in the morning, midday, afternoon, and evening | 16 minutes | 4 | 14 |
| van Knippenberg, 2018 [47] | • One item (down) among 8 NA  • Self-reporting question based on 7-point Likert scale | • Self-esteem  • Physical well-being  • Current context (social company, activities, location, and important events)  • PA (cheerful, relaxed, enthusiastic, and satisfied)  • Other 7 NA (insecure, lonely, anxious, irritated, desperate, tensed, and confident) | • Palmtop with application (PsyMate) | • Random basis from 7am30 to 10pm30 | Not described | 10 | • Baseline: 3  • Intervention: 18 (3 consecutive days * 6 cycles)  • Post-intervention: 3 |
| Elliston, 2017 [19] | • One item (sad) among 6 NA  • Self-reporting question based on 0–100 slider scale | • Momentary food environment  • External cues (others eat, food availability, with others)  • Other 5 NA (angry, bored, irritable, stressed, and restless)  • Food reports | • Smartphone | • Mixed: (1) Random basis at 3-5 times per day  (2) Whenever eating or in the evening | Not described | 3-9 | 14 |
| Forman, 2017 [20] | • One item (sad) among 5 NA  • Self-reporting question based on 5-point Likert scale | • Time of lapse  • Lapse type  • Lapse location  • Presence of tempting foods  • Non-affective status (hunger, perceived deprivation, and tiredness)  • Other 4 NA (lonely, bored, angry/irritated, and stress) | • Smartphone with custom application (DrexelEMA) | • Mixed: (1) Quasi-random basis at 6 times per day  (2) Whenever they experienced a dietary lapse | Not described | At least 6 | • Baseline: 14  • Mid-treatment: 7  • End-of-treatment: 7 |
| Liao, 2017 [21] | • One item (sad/depressed) among 4 NA  • Self-reporting question based on 5-point Likert scale | • Current activity  • Company of a dog  • PA (happy, cheerful, and calm/relaxed)  • Other 3 NA (nervous/anxious, stressed, and frustrated/angry) | • Mobile phone with application | • Quasi-random basis within 2 hours block from 6am30 to 10pm | Not described | 6 among 8 possible | 12 (4 consecutive day * 3 cycles) |
| Maisto, 2017 [22] | • One item (sadness) among 4 affect states  • Self-reporting question based on 7-point Likert scale | • Stress  • Social context  • Cognitive variables  • Other 3 affect states (anger, fear, and happiness) | • Cellular phones with Interactive Voice Response (TeleSage, SmartQ) and a computer telephone voice board (Dialogic) | • Quasi-random basis with a 4 hours block | Not described | 4 | 28 |
| Verhagen, 2017 [23] | • One item (down) among 9 NA  • Self-reporting question based on 7-point Likert scale | • Context items  • Important events  • Somatic complaints  • Physical condition  • Levels of beep disturbance  • Quality of sleep  • Average mood  : PA (cheerful, satisfied, relaxed, and globally feeling well)  : Other 8 NA (lonely, guilty, worried, threatened, insecure, irritated, frightened, and suspicious) | • Smartphone with application (PsyMate) | • Quasi-random basis with 1.5 hours block from 7am30 to 10pm30 | Not described | 10 | 12 (6 consecutive days * 2 cycles) |
| Depp, 2016 [9] | • One item (sad or depressed) among 7 affective ratings  • Self-reporting question based on 7-point VAS | • Momentary impulsivity  • Daily life activities  • Location  • Social context  • Other 6 affective ratings (happy, energetic, angry or upset, anxious or nervous, stressed, and relaxed) | • Smartphone | • Quasi-random basis within two 3-4 hours block in the morning and evening | 2 hours | 2 | 77 |
| Eldahan, 2016 [10] | • One item (depressed) among 6 NA  • Self-reporting question based on 4-point Likert scale | • Daily minority stress  • PA (excited, alert, determined, enthusiastic, and inspired)  • Other 5 NA (sluggish, distressed, upset, discouraged, and stressed)  • Anxious affect (jittery, scared, afraid, anxious, and nervous) | • Online daily diary via daily email link  (unclear of the device) | • Fixed basis at 8pm | 14 hours | 1 | 30 |
| Paterson, 2016 [44] | • One item (sad) among 9 emotional outcome (PA and NA)  • Self-reporting question based on 101-point VAS | • Coping  • Social support  • Other 8 emotional outcome (PA and NA) (tired, alert, happy, nervous, frustrated, stressed, energetic, and angry) | • PDA with electronic behavioral diary | • Fixed basis at 3 intervals per day at pre-determined by individual | Not described | 3 | 31 |
| Ramsey, 2016 [34] | • Depression  • Single self-reporting question based on 5-point Likert scale | • Anxiety  • Mindfulness | • Smartphone | • Random basis | Not described | 3 | 20 (10 consecutive days * 2 cycles) |
| Ravesloot, 2016 [6] | • Depressed mood  • Single self-reporting question based on 5-point Likert scale | • Pain  • Fatigue  • Their location and experience | • Tablet computers  • Global Positioning System of tablet computers | • Quasi-random basis within 2 hours block from 9am to 9pm | Not described | 6 | 14 |
| Smith, 2016 [36] | • Two items (depressed and sad) among 5 NA  • Self-reporting question based on 5-point Likert scale | • Fatigue  • Pain  • PA (happy, content, warm toward others, interested, and energetic)  • Other 3 NA (frustrated, irritated, and worried) | • Telephone call for brief interview | • Quasi-random basis within 3 hours block over a 12 hours period pre-determined by individual | Not described | 4 | 7 |
| Vachon, 2016 [7] | • One item (depressive mood) among 7 affective and cognitive states related to depression  • Self-reporting question based on 11-point VAS | • Other 6 affective and cognitive states related to depression (self-esteem, physical-self, quality of life, coping, rumination, and anxiety) | • Computerized device | • Fixed basis in a 12-hour block pre-determined by individual | 2 hours | 2 | 6-month |
| Vasconcelos e Sa, 2016 [24] | • One item (sad) among 6 NA  • Self-reporting question based on 7-point Likert scale | • Psychosis experiences (only assessed in patients)  • Dyad contact  • Dyad behaviorally controlling interactions  • PA (happy, cheerful, satisfied, excited, and relaxed)  • Other 5 NA (guilty, irritable, anxious, annoyed, and lonely) | • Digital wristwatch (alarm)  • Palm device with ESP^f^ software (record) | • Random basis from 9am to 12am | 15 minutes | 10 | 6 |
| Burns, 2015 [25] | • One item (sad) among 6 NA of patient  • Self-reporting question based on 9-point Likert scale | • Patient-reported  : Trait and state of pain catastrophizing  : Pain-related variables  : Spouse criticism, hostility, and support  : Other 5 NA (anxious, on edge, uneasy, helpless, and discouraged) of patient  • Spouse reported  : Observed patient pain-related variables  : Criticism, hostility directed toward patient | • PDA with ESP completed electronic daily diary | • Fixed basis at 8am50, 11am50, 2pm50, 5pm50, and 8pm50 | 15 minutes | 5 | 14 |
| Droit-Volet, 2015 [48] | • One item (sad) among 4 affective states  • Self-reporting question based on 7-point Likert scale | • Passage of present time  • Arousal level  • Attention to current activities  • Among others  • Pain  • Other 3 affective states (happy, excited/stimulated, and relaxed/calm) | • Smartphone (alarm)  • Writing in a small booklet or checking scales (record) | • Quasi-random basis with approximately 1.5 hours block from 8am to 8pm | Not described | 8 | 5 |
| Dunton, 2015 [26] | • One item (sad/depressed) among 4 NA  • Self-reporting question based on 5-point Likert scale | • Activity level and intensity  • Social and physical context  • PA (happy, cheerful, and calm/relaxed)  • Other 3 NA (nervous/anxious, stressed, and frustrated/angry) | • Mobile phone with a custom version of the MyExpereince software  • Waist-worn accelerometer | • Random basis from 6am30 to 10pm | 15 minutes | 60% among 8 possible | 12 (4 consecutive day * 3 cycles) |
| Floridou, 2015 [27] | • One item (happy/sad) among 6 mood states • Self-reporting question based on 7-point Likert-type scale | • Occurrence or not of INMI^g^ • Mind wandering episodes at the time of the prompt • Current activity  • Details of the INMI experience • Details of the mind wandering content  • Other 5 mood states (alert/drowsy, tense/relaxed, interested/bored, energetic/tired, lonely/connected) | • Text message of mobile phone (alarm)  • Experience Sampling Booklet (record) | • Random basis from 8am to 11pm | Not described | 6 | 7 |
| Epler, 2014 [28] | • One item (sadness) among 2 NA  • Self-reporting question based on 5-point Likert scale | • Hangover  • Likelihood of drinking tonight  • Drinking events  • Stressors  • Subject states  : Crave a drink  : Physical effects (sluggish, buzzed, dizzy, headache, and nauseous)  : PA (enthusiasm, excited, and happy)  : Other 1 NA (distress) | • Palmtop computers completed electronic diary | • Mixed: (1) Once at wakeup time (2) Whenever follow-up drinking events | 3 minutes | At least 1 | 21 |
| Mazure, 2014 [29] | • One item (sad mood) among 7 depressive symptoms  • Self-reporting question based on 7-point Likert-type scale | • Stressful daily life events  • Perceived stress  • Other 6 depressive symptoms (anhedonia, fatigue, concentration difficulties, appetite change, negative thoughts, and hopelessness) | • PDA with modified version of the Purdue Momentary Assessment Tool, version 2.1.2. | • Quasi-random between 9am-11am, 11am -2pm, 2pm-5pm, 5pm- 8pm, 8pm-10pm | 15 minutes | 5 | 7 |
| Ram, 2014 [30] | • Negative feeling (sad) at the end of the day  • Single self-reporting question based on 101-point sliding bar scale | • Social interactions  : Thoughts  : Behaviors  : Positive feeling (happy) | • Smartphone with application (iSAHIB^h^ Survey) | • Unclear | Not described | 1 | 63 (21 consecutive day * 3 cycles; spaced at 4.5 months interval) |
| Wolf, 2014 [31] | • Depressive symptoms  • Five self-reporting question based on 3-point Likert scale | • Pain  • Loneliness  • Occurrence of positive and negative interpersonal events  • Appraisal of interpersonal event | • Automatic calls to cell phone completed electronic diary | • Mixed: (1) Once at morning call time occurred 30 minutes after usual wake time  (2) Fixed basis at 11am, 3pm30, and 7pm | 3 hours | 4 | 21 |
| Scott, 2013 [32] | • One item (sad) among 3 NA  • Self-reporting question based on 5-point Likert scale | • Stress  : Stressor event  : Stressor severity  : Global perceived stress  • PA (happy, excited, and alert)  • Other 2 NA (nervous and irritated) | • PDA | • Fixed basis at 9am 12pm, 3pm, 6pm, and 9pm | 30 minutes | 5 | 10 |
| Kööts, 2011 [49] | • One item (sad) among 7 basic emotions  • Self-reporting question based on 4-point Likert scale | • Whereabouts  • 5 other emotion-related adjectives (disappointed, in physical pain, irritated, sleepy, and tired)  • Other 6 basic emotions (anger, happy, contempt, disgust, fear, and surprise) | • Palmtop computer with iESP software | • Random basis from 8am to 8pm | Not described | 7 | 14 |
| Piasecki, 2011 [33] | • One item (sad) among 2 NA  • Self-reporting question based on 5-point Likert scale | • Alcohol and cigarette use  • Appraisals of alcohol and cigarette effects  • Occasion-level covariates (others present and location)  • Current subjective states  : PA (enthusiastic, excited, and happy)  : Other 1 NA (distressed)  : Craving (crave a cigarette and drink)  : Possible effects of alcohol intoxication and alcohol withdrawal/hangover (buzzed, dizzy, sluggish, headache, and nauseous) | • Palmtop computers completed electronic diary | • Mixed: (1) Random basis (2) Whenever individualized based on smoking and drinking | 3 minutes | Up to 5 | 21 |
| Hachizuka, 2010 [45] | • Depression  • Single self-reporting question based on 101-point VAS | • Pain  • Fatigue  • Nausea  • Anxiety  • Drowsiness | • PDA completed electronic diary | • Mixed: (1) Quasi-random basis in once in the morning and once in the afternoon  (2) When the patient took their usual analgesics (one to three times per day)  (3) Whenever patient took rescue medications and at 30, 45, 60, and 90 minutes later in sequence | Not described | At least 3 | 7 |
| Poulin, 2010 [41] | • One item (depressed/ blue) among 7 NA  • Self-reporting question based on 5-point Likert scale | • Active helping time  • On call time  • PA (happy, joyful, pleased, and enjoyment/fun)  • Other 6 NA (unhappy, frustrated, angry/hostile, worried/anxious, guilty, and stressed) | • PDA | • Quasi-random basis with approximately 3 hours block during waking hours | Not described | Unclear | 7 |
| Dunton, 2009 [42] | • One item (sad/depressed) among 7 NA  • Self-reporting question based on 10-point Likert scale | • Moderate-to-vigorous physical activity  • Self-efficacy  • Control and demand  • Fatigue  • Energy  • Social interactions  • Stressful events  • PA (happy)  • Other 6 NA (emotionally upset, stressed, lonely/alone, annoyed/angry, tense/anxious, and discouraged/frustrated) | • Handheld computer  • Handheld electronic diaries | • Fixed basis at 7am45, 11am45, 3pm45, and 7pm45 | 45 minutes | 4 | 14 |

^a^EMA: Ecological Momentary Assessment.

^b^NA: Negative Affect.

^c^PA: Positive Affect.

^d^VAS: Visual Analogue Scale.

^e^PDA: Personal Digital Assistant.

^f^ESP: Experience-Sampling Program.

^g^INMI: Involuntary Musical Imagery.

^h^iSAHIB: Intraindividual Study of Affect, Health and Interpersonal Behavior.
