# Supplementary material for: Possible Application of Ecological Momentary Assessment to Older Adults’ Daily Depressive Mood: Integrative Literature Review
Source: JMIR Ment Health. 2020 Jun 2;7(6):e13247. doi: 10.2196/13247 (PMC7298638; doi:10.2196/13247)
Supplement: Multimedia Appendix 4 [file mental_v7i6e13247_app4.docx]

Multimedia Appendix 4. Definitions and rates of dropout, adherence, and compliance used in the selected studies.^a-g^

| First author,  year | Definition | Rate or usage reported |
| --- | --- | --- |
|  |  |  |
| Crowe, 2019 [13] | Poor compliance = answering less than 30% of reports per day over at least 2 days | - Dropout = 3.1% (2/64) - Among 2,548 momentary surveys collected, exclusion criteria resulted in a loss of 313 reports (117 control and 196 MDD^a^ reports). - MDD participants completed an average of 33.1 valid ESM^b^ questionnaires per individual, compared to an average of 36.5 for controls. |
| de Hoog, 2019 [14] | Poor compliance = answering less than 17 of the 50 times (1/3 of the possible entries required to produce a valid data set in ESM studies) | - Dropout = 25.9% (22/85) - Participants are most compliant at the start of the study with decreasing pattern within days or over the time. |
| Mogle, 2019 [15] | Dropout = those who did not complete the primary measure of interest daily | - Dropout = 12.8% (23/180) - Compliance rate = 82.7% - Of 1,162 possible assessments, participants completed 996 assessments. |
| Hooker, 2018 [39] | Not described | - Compliance rate = 75.5–80.9% - Each participant completed a median of 24 of the 28 daily surveys with possible range between 2 and 28. |
| Jamison, 2018a [43] | Dropout = those who expressed withdrawal from the study | - Dropout = 7.2% (5/69) - The total number of daily assessments from the pain app over three months (M^c^ = 60.6, SD^d^ = 29.6, range = 0–106). |
| Jamison, 2018b [16] | Dropout = those who expressed withdrawal from the study or died | - Dropout = 13.3% (12/90) - Compliance rates = 70% for the three-month and 55.1% completed the six-month evaluation. - The total number of daily assessments were averaged 69.3 (SD = 71.8) over an average of 110.5 (SD = 80.7) days. |
| Kuerbis, 2018 [46] | Not described | - Compliance rates = Total (morning 87.5%, evening 77.2%); Adults < 49 (morning 86.3%, evening 72.4%); and Adults > 50 (morning 89.6%, evening 86.3%) - Factors of high compliance rates: morning survey, older drinkers on the evening survey, and smartphone users. |
| Lamers, 2018 [17] | Not described | - Compliance rates = 78.5% - Each participant completed 44 (SD = 10.0) out of 56 electronic assessments (range 3–56, median 47, IQR^e^ 40–51 - 60.3% of the sample completed 80% of the assessments. |
| Neubauer, 2018 [18] | Not described | - Dropout = 2.8% (5/180) - Compliance rates = 92.8% (Burst 1), 94.1% (Burst 2), and 93.2% (Burst 3) - 102 participants completed all three bursts. |
| Paolillo, 2018 [40] | Not described | - Participants completed an average of 89.5% of possible EMA^f^ surveys (M = 49.4, SD = 5.9, range = 31–56). |
| van Knippenberg, 2018 [47] | Poor compliance = answering less than 33% of the total 30 ESM reports | - Dropout = 22% (11/50) - Compliance = 76.4% (M=137.4, SD = 20.2 out of 180) |
| Elliston, 2017 [19] | Poor compliance = answering less than 50% of random prompts | - Compliance = 95.3% - Each participant completed an average of 14 days of monitoring (M = 14.61, SD = 1.46). - On average, participants missed 0.12 random prompts (SD = 0.37) per day. |
| Forman, 2017 [20] | Poor compliance = answering less than 40% compliance at a given assessment point | - Compliance = 98.4% (baseline n = 186/189), 99.4% (mid-treatment n = 162/163), 97.2% (end-of-treatment n = 143/147)   -Baseline: M = 83.2%, SD = 11.5, range = 45–100  -Mid-treatment: M = 82.7%, SD = 14.7, range = 42–100  -End-of-treatment: M = 85.5%, SD = 13.2, range = 42–100   - 138 individuals completed EMA at all three assessment points with ≥ 40%. |
| Liao, 2017 [21] | Not described | - Dropout rate = 17.1% (wave2) and 19.7% (wave 3) - The person-level EMA response rate ranged from 46.3% to 100% (M = 82.7%, SD = 0.11). On average, the response rate was 80.7% (SD = 0.15) in the morning, 84.2% (SD = 0.119) in the afternoon, and 83% (SD = 0.15) in the evening. |
| Verhagen, 2017 [23] | Poor compliance = answering less than ten beeps during a measurement period (either baseline or follow-up) | - Compliance rate = 85.3% (64/75) - At baseline, the response percentage was 52.7% of 3780 presented beeps, comparable to the 48.2% of 1740 presented beeps at follow-up. |
| Depp, 2016 [9] | Adherence = number of days of survey completed/total number possible | - Adherence = 65.1% (SD = 0.22) - The 41 participants submitted a total of 2,902 survey epochs over a maximum of 77 days (M = 50.1days, SD = 17.6, range = 12–76). |
| Eldahan, 2016 [10] | Dropout = those did not complete any daily diaries | - Dropout = 1.3% (5/376) - This represents a median of 25 (M = 22.3) days of completion per participant or median adherence of 83.3% (M = 74.3%). |
| Paterson, 2016 [44] | Not described | - Dropout = 8.3% (1/12) - Majority of response rates were > 90%. |
| Ramsey, 2016 [34] | - Acceptable adherence = at least 30% of total surveys completed based on the pre-established standard set by Rullier et al. [35] - Perfect adherence = all EMA completed over the study period | - 76% of participants completed one or more surveys on at least 10 different days. - Acceptable adherence = 70% - Perfect adherence = 46–48%: All participants missed at least one assessment, although 18% of participants at Time 1 and 16% of participants at Time 2 self-reported perfect adherence. |
| Ravesloot, 2016 [6] | Adherence = at least half of the scheduled EMA prompts over the study period | - Dropout based on adherence criteria = 6.7% (10/149) - Compliance rate = 87%: Respondents completed 87% of the 84 possible study prompts. |
| Vachon, 2016 [7] | - Dropout = lack of compliance during the follow-up less than 50% - Compliance rate = dividing the number of completed ratings by the potential maximum number of self-evaluations | - Dropout = 20% (6/30) - Compliance rate = 81.2% (SD = 13.9%) |
| Vasconcelos e Sa, 2016 [24] | Compliance = answering more than 20 (out of possible 60) valid ESM reports | - Both patients and relatives provided data on average at over half of the sixty assessments (M = 40.1, SD = 10.6; M = 45.4, SD = 7.9, respectively). |
| Burns, 2015 [25] | Not described | - Compliance = 80–87.1% out of the 7350 of possible total responses - For patients and spouses, 87.1% and 89.1% of records were complete, respectively. |
| Droit-Volet, 2015 [48] | Not described | - Dropout = 3.4% (1/29) - A mean percentage of missing alerts of 8% (SD = 6.8, minimum = 0, maximum = 22). |
| Dunton, 2015 [26] | Not described | - Compliance = 83% - Respondents completed 7,910 Level 1 observations (M = 68.19, SD = 22.2, range = 10–96 per participant). - Factors of high compliance rates: weekdays, afternoon low BMI^g^ score or waist circumference. |
| Floridou, 2015 [27] | Not described | - Dropout = 5% (2/40) - Compliance rate = 86% (1374 out of the total of 1596 prompts) |
| Mazure, 2014 [29] | Dropout = those reported less than 6 valid assessments based on 5 times a day of EMA | - Dropout = 10.4% (5/48) - 43 participants responded to 83.7% of EMA or a mean of 29 out of the 35 possible assessments (SD = 17.4). |
| Ram, 2014 [30] | Not described | - Dropout = 9.3% (14/150): Of the 150 participants, 136 (90.7%) completed the entire intensive protocol, and 14 withdrew after completing between a third (n = 11; 7.3%) and two-thirds (n = 3; 2%) of the total protocol. |
| Wolf, 2014 [31] | Not described | - Dropout = 23.4% (36/154) - Compliance rate = 80.4%: Participants completed 16.89 of 21 possible days (range = 1–23, SD = 5.2). |
| Scott, 2013 [32] | Compliance = participants who responded to 80% of the beeps | - Compliance rate = 79% of surveys within 30 min of the beep prompt |
| Kööts, 2011 [49] | Not described | - Compliance rate = 82.8% (8,835/10,667 possible) - The number of usable trials per participant ranged from 37 (of 49 possible) to 95 (of 98 possible) (M = 80.32, SD = 10.6). |
| Piasecki, 2011 [33] | Not described | - Compliance rate = 78.8% - Overall, smokers logged a total of 16,670 smoking events and 6,617 (39.7%) were followed by a full assessment. |
| Hachizuka, 2010 [45] | - Overall compliance rate = averaging the response rate over all subjects - Individual compliance rate = dividing the number of prompts completed by the number of prompts scheduled | - Dropout = 16.7% (3/18) - The response rate was 80.2% (SD = 16.8) for taking rescue medications; 90.3% (SD = 10.5) for scheduled recordings on average. |
| Dunton, 2009 [42] | Not described | - Compliance rate = 87% - Average of 3.59 (SD = 0.85) diary entries per day summing to a total of 983 diary entries (out of 1,288 diary prompts). |

^a^MDD: Major Depressive Disorder.

^b^ESM: Experience Sampling Method.

^c^M: Mean.

^d^SD: Standard Deviation.

^e^IQR: Inter Quartile Range.

^f^EMA: Ecological Momentary Assessment.

^g^BMI: Body Mass Index.
